# Chloroform in Convulsions of Infants and Children

**Published:** 1879-06

**Authors:** J. D. Blake

**Affiliations:** Baltimore, Md.


### ARTICLE V.

### *Chloroform in Convulsions of Infants and Children.*

BY J. D. BLAKE, M. D., BALTIMORE, MD.

Certain it is, that we are called upon to treat few diseases that are more formidable or more fatal than convulsions of infants and children. The great number of deaths from convulsions, especially of infants, which we constantly find in all of our mortality returns, I think will bear witness to the above statement, for correctness. Not desiring, however, to enter to any great extent upon the question of the nature of the different types or forms of convulsions of early life, I shall content myself at this time with referring to the general opinion of pathologists of the present day, that by far the greatest proportion of infantile convulsions are sympathetic or functional merely. "The grand reason (says Dr. West) of their frequency is, no doubt, to be found in the preponderance or predominance of the spinal over the cerebral system in early life. Thus it is, that in a large majority of cases of convulsions we find the cause due to some morbific agent acting upon some distant excitant surface or part, as the teeth, stomach, bowels, etc., there being, as a rule, no organic lesions found after death." Consequently, in cases of infantile convulsions, particularly when of a sympathetic, reflex or eccentric type, after removing all traceable exciting source of irritation, and diminishing as far as possible any excess of vascular action in the nervous centres, physicians have generally proceeded to combat the disease, if it still persisted, with medicinal agents, that tended to reduce the super-irritability of the excito-motory system, or otherwise to restore it to a proper and healthy standard of action.

To fulfill this indication, preparations of different kinds have been used, such as chloral, bromide of potassium, the different preparations of opium, hyoscyamus, and other antispasmodics too numerous to mention. And yet, notwithstanding these and the long list of other preparations used and recommended by physicians, we are constantly called to cases of infantile convulsions over which these remedies seem to exert but little, if any, control; indeed, in a large number of cases we find it an impossibility to administer any medicine, whatever, from the continued muscular jerking and spasm of the jaw. In such cases it is customary to look to the condition of the skin, bowels, head, etc.: thus we use enemata, warm baths, cold to the head if indicated, and sometimes when not. This is all that is left us to do, and this, in many instances, rather to relieve the great anxiety of the parents and relatives, than the condition of the patient. There is no doubt that there is a goodly number of cases relieved or moderated by such remedies as above spoken of. But these are not the cases of which I wish to speak at this time. It is of those cases which we find do not yield to such remedies; in other words, the chronic or subacute form of convulsions, which, on account of their duration, exhaust the little patient to such an extent as to prevent its recovery, even if it should outlive the convulsions.

In order that I may more fully explain myself, I shall relate a few cases, selected from fourteen, in which I have taken special note, and in which I have used chloroform with so much benefit to my little patients and satisfaction to myself.

Case 1.—March 13th, 1877, I was called, about 3 P. M., to see a little son of Mr. K., eight months old. When I arrived at the home of my patient I found him in violent convulsions, affecting, at that time, both sides, at other times only one side. Upon inquiring, I was informed by the mother that Dr. I., their family physician, had been called early in the day, and had prescribed for the patient, gave it an enema and a warm bath, after which he left, saying that the convulsions would soon cease. The Doctor was giving one-twelfth grain of calomel, with one grain of sugar, every half hour. In addition to this, he ordered potassium bromide and chloral, with tincture of hyoscyamus and syrup, to be given in half teaspoonful doses every hour, if the clenched teeth or convulsion did not prevent, which they did, consequently none of the medicine could be given. Finding the convulsions did not cease, the mother gave it a mustard bath, applied mustard to the spine, feet, hands, legs, etc. Notwithstanding all this the convulsions continued, more violent at times than at others. The patient at this time was bathed in perspiration; pulse so rapid that to count it was impossible; pupils dilated. Knowing that he was becoming rapidly exhausted, I immediately ordered half an ounce of Squibb's chloroform, which I allowed him to inhale from my handkerchief until he was perfectly relaxed and the convulsive movement had entirely ceased, which I do not think took more than two minutes from the time I commenced the inhalation. The pulse now became more regular and full, the breathing was calm and easy, and the child passed into a sweet sleep, from which it did not awake for thirty-six minutes, at which time I noticed a slight twitching of the muscles of the face and eye. I again applied the handkerchief, only allowing him to take an occasional whiff, when he again fell into a quiet sleep, and remained so for three-quarters of an hour. In the meantime Dr. I. arrived, and seemed somewhat surprised to find that his prescriptions had failed to relieve the child, but expressed satisfaction with the result of chloroform in the case. He said he had never used it in such cases, but thought he would in future when such cases presented themselves. I then resigned the patient to Dr. I., who told me next morning that he had no further trouble. The patient, he said, awoke much refreshed, took a little nourishment and went off to sleep again, from which he did not awake for two hours. He made a rapid recovery.

Case 2.—On the night of June 17th, 1878, I was summoned in great haste to see a fourteen months old daughter of Mr. L. B., who, I was informed upon arriving, had been in convulsions since early in the afternoon; their family physician, who was a homœopath, had used all of his remedies without benefit; in addition, the child had been put in a warm mustard bath, an enema had been given, the bowels had moved freely, it had vomited after an emetic had been given, and yet the convulsions continued, and at times very violent; so much so that the physician gave the case up as hopeless, saying it could not recover. I used chloroform here as in the other case, with almost equal success. The child having been placed thoroughly under the influence of the anæsthetic, I found the pupils, which were widely dilated, had contracted down almost to the normal condition; the pulse, which was thread-like and so quick that I could not count it, became full and much slower; the respiration became slower and more natural. In this case however, I had to renew the inhalations three times, as the convulsive movements would return as the effect passed away. On the third application of the inhaler, however, the child remained asleep for more than an hour, when it awoke, with no return of the convulsions, took a little water, went off to sleep again, arousing two and a half hours later. With the exception of being restless and a little fretful, she made a rapid recovery. Now, there are many other cases that I might relate, but this I think will suffice to prove the efficacy of chloroform in such cases; and especially so, as all the usual remedies had been used without success.

There is another class of cases in which I think chloroform serves both to control the convulsive movements and aid materially in making our diagnosis. The following case will illustrate my meaning: I was called January 2d, about 1 o'clock, A. M., to see a nine year old son of Mr. L., who was taken with convulsions about mid-night. The cause was unknown, as he had never had convulsions before. I ordered an enema, also a warm bath, in addition to one he had received before my arrival. The bowels moved freely; the convulsions continued without the slightest abatement; I could get nothing into his mouth. I ordered chloroform, which I used as before, always keeping up the inhalation until the convulsive movements ceased entirely. When this took place, however, in this patient, I had the satisfaction of seeing him become excessively nauseated, followed by copious vomiting. Upon examination of the matter vomited, I found it to contain large quantities of *peanuts*, pieces of candy, and undigested food, which the boy had obtained the evening before by visiting his grandparents without the knowledge of his parents. After the stomach was evacuated the boy had no more convulsions, but was able to get up and play around the next morning. Now, had I known the cause of the convulsions, or the contents of the stomach, I know of no means by which I could have administered an emetic which would have acted so promptly, so easily and so satisfactorily as did the chloroform.

It is a well known fact that chloroform possesses the property of diminishing the power of the nerves in receiving and communicating, and of the brain in perceiving sensation, whether arising from internal or external causes; it further arrests the reflex action of the spinal cord. This being its action, we do not think the day far distant when chloroform will be universally recognized as the remedy par excellence in convulsions of infants and children.—*Med. and Surg. Reporter.*

---

